# Importance of neuropsychological screening in physicians referred for performance concerns

**DOI:** 10.1371/journal.pone.0207874

**Published:** 2018-11-26

**Authors:** Betsy White Williams, Philip Flanders, Dillon Welindt, Michael V. Williams

**Affiliations:** 1 Department of Psychiatry School of Medicine, University of Kansas, Clinical Program, Kansas City, Kansas, United States of America; 2 Professional Renewal Center (PRC), Lawrence, Kansas, United States of America; 3 Wales Behavioral Assessment (WBA), Lawrence, Kansas, United States of America; Lluita contra la SIDA Foundation - Germans Trias i Pujol University Hospital - Autònoma de Barcelona University, SPAIN

## Abstract

**Introduction:**

The literature suggests that 6–12% of practicing physicians are dyscompetent. Dyscompetence can manifest as failures in direct provision of care, but also issues with interpersonal and communications skills and professionalism. There is a growing literature suggesting the value of neurocognitive screening in physicians with clinical competency issues. The contribution of such screening in physicians with workplace behavioral issues is not as established. The aim of this exploratory study was to examine patterns of performance on a commonly used neuropsychological screening instrument. Performances differences, if present, could have implications for remediation and/or monitoring.

**Methods:**

Published data on a computerized neurocognitive screening instrument (MicroCog) for normative physician samples, published data on physicians referred for clinical competency issues, and newly collected data on physicians with workplace behavioral issues were analyzed. A two-way analysis of variance (Sample X Index) and post-hoc paired comparisons were conducted. A second analysis was performed employing an aggregated estimate of normative physician performance.

**Results:**

Results revealed a significant main effect for Sample and Index and a significant interaction effect. The second analysis of variance employing the pooled samples (Sample X Index) was conducted. The workplace behavior issues sample differed significantly from each of the samples. The Sample by Index interaction was significant.

**Discussion:**

Significant differences in performance on a neurocognitive screening instrument were found between non-referred physicians and physicians with behavioral or medical/technical competency concerns. Those with workplace behavioral issues performed significantly better than those with medical/technical issues, but significantly worse than non-referred physicians. Using these findings, 2.0% of the normal sample versus 35.1% of the medical/technical sample, and 10.9% of the behavioral sample would fail the screen using typical, conservative cutoffs. Further study of the potential role of neurocognitive factors in physicians referred for behavioral comportment issues is warranted.

## Introduction

Physicians have enormous responsibilities to the community. To prepare physicians to fulfill those responsibilities, they participate in a highly competitive and selective admissions process and a rigorous course of education, training, and testing for proficiency. Not all physicians maintain their level of knowledge and currency [1]. Doctors who fail to maintain acceptable standards in one or more areas of professional practice are referred to as dyscompetent [2]. The term *underperformance* is a broader term and refers to a level of performance that is below expectation[3]. While the exact number of dyscompetent and underperforming physicians is not known, the literature suggests that estimates for dyscompetence range from 6%-12%, with additional physicians classified as underperforming [3].

The concern for underperformance and/or dyscompetence among practicing physicians has resulted in increased efforts to ensure ongoing competence of physicians. This appreciation is reflected in rules and procedures implemented by various organizations across the physician career-span to ensure the competence of licensed physicians. Efforts have included mandatory completion of continuing medical education (CME), Maintenance of Certification (MOC), Ongoing Practice Performance Evaluations/Focused Practice Performance Evaluations (OPPE/FPPE), and revalidation [4–10].

Underperformance and dyscompetence do not just apply to issues of cognitive and technical competence. The American Board of Medical Specialties (ABMS) and the Accreditation Council of Graduate Medical Education (ACGME) have identified six competency areas as core to medical performance: patient care, medical knowledge, practice-based learning and improvement, interpersonal and communication skills, professionalism, and systems-based practice. Dyscompetence in any of these areas can contribute to issues of lowered patient safety and reduced quality of patient outcomes [11–17].

The importance of areas beyond medical knowledge and technical skills as critical for the delivery of the highest quality health care has been documented in countries beyond the United States. Revalidation is the process by which the General Medical Council requires licensed doctors to demonstrate they are up to date on an ongoing basis and fit to practice. The General Medical Council[5], for example, notes that in order to be revalidated, each doctor must meet requirements in the areas of knowledge, skills and performance; safety and quality communication, partnership and teamwork; and maintaining trust, while the Royal College of Physicians and Surgeons of Canada advocates for the creation of a learning culture characterized by practice reflection, inquiry, peer review and rigorous formative assessments of knowledge (through self-assessment programs), competence (through simulations) and performance (through practice reviews) that reflect the entire spectrum of roles and competencies associated with the CanMEDS framework [4].

A number of factors have been demonstrated to contribute to performance difficulties. These include issues associated with age, medical health, psychiatric health, personality characteristics, attitudes/beliefs, life stressors, burnout, developmental stressors, system issues, poor initial preparation, and failure to maintain currency [16]. Some of these issues can lead to concerns about impairment. The AMA has defined physician “impairment” as “the inability to practice medicine with reasonable skill and safety due to 1) mental illness 2) physical illnesses, including but not limited to deterioration through the aging process, or loss of motor skill, or 3) excessive use or abuse of drugs, including alcohol.” Studies suggest that physicians referred to programs that assess and remediate medical/clinical competence issues perform significantly worse on neuropsychological testing than their peers[12, 18–22] and that cognitive impairments likely contribute to competency issues and failure to improve with remedial CME [12, 20].

Given the diverse potential contributory factors to physician performance issues, an assessment informed by a broad model is required to diagnose and remediate performance issues. An exemplar of this is a biopsychosocial model (BPS) that conceptualizes health as the interaction among biological, psychological, and social factors [23]. An effective intervention, therefore, would involve an approach synthesized from determining needs assessed by a broad review of all areas. This study primarily focuses on the biological and psychological aspects of the model. One method for gaining insight into possible health concerns is the assessment of neurocognitive functions. Assessing neuropsychological performance in physicians, however, can be challenging as measures of the intellectual performance of US physicians on standardized tests of intelligence have found that medical students’ and physicians’ intellectual performance is approximately one to two standard deviations above the mean of the population [24, 25]. A recent report on the best evidence for norms for physicians on the MicroCog, a commonly employed neuropsychological screening tool, has documented the need for different norms for physicians than the current age and education adjusted norms reported in the MicroCog manual [26].

Proper evaluation and identification of the causes of performance deficiencies is critical for determining appropriate recommendations, including steps for remediation, necessary oversight and the need for additional evaluations [27]. While the evidence cited is suggestive of the potential contribution of biological and psychiatric conditions in physicians in whom there are clinical competency concerns, it remains to be demonstrated if such conditions have a potential contributory role in the performance of physicians referred for behavioral issues that include performance difficulties within the core competency areas of interpersonal and communication skills, professionalism, systems-based practice, and practice-based learning and improvement. We refer to issues in these four core competency areas as behavioral comportment issues.

The aim of this exploratory study was to examine patterns of performance on a commonly used neuropsychological screening instrument. Performances differences if present could have implications for remediation and/or monitoring. Given our clinical experience that a high percentage of referred physicians have health issues that could impact their functioning[28] and the previous literature on neuropsychological issues in physicians with medical technical concerns, we hypothesized that physicians with behavioral comportment issues would perform significantly worse on a frequently used neurocognitive screening instrument than published data on comparison physicians. However, as referral sources had not raised concern about elements of clinical competency in the physicians referred for behavioral comportment issues, we were uncertain how those with behavioral comportment issues would perform in comparison to those with medical/technical concerns.

## Methods

### Study data and samples

This study employed a design using publicly available data from prior published reports of physician performance on the MicroCog along with a new sample of previously collected de-identified data. In all, data from five sources were analyzed: one from the general population, two from normal physicians, and two from physicians referred for evaluation secondary to concerns about elements of their performance. More specifically, the five samples included: Sample 1) Age and education corrected norms for the MicroCog as reported in the manual (MicroCog Norm sample)[29]; Sample 2) a physician sample reported in the MicroCog manual (Powell sample)[30]; Sample 3) published findings from the previously mentioned study of a physician control sample (Korinek Control sample)[31]; Sample 4) published findings from a study of underperforming physicians with medical/technical concerns referred for clinical competency evaluation to a non-for-profit center (Korinek Medical/Technical sample)[31]; and Sample 5) new data from physicians referred to a Midwestern center for assessment and remediation of workplace behavioral issues (Williams Behavioral Comportment sample). The Korinek Medical/Technical sample, beyond identifying the physicians as having been referred for assessment of their competence secondary to performance reviews, does not provide information on the types of competency concerns [19]. Clinical competence evaluations typically have more of a focus on issues related to medical knowledge, medical judgment, clinical decision-making, procedural skills and poor charting among other skill sets. Clinical competency assessment elements often include completion of standardized tests, participation in structured clinical interviews, chart reviews, procedural simulation and neurocognitive screening. A neurocognitive screening assessment is also typically included as part of assessment process. Physicians in the Williams Behavioral Comportment sample were referred from across the United States for fitness for duty evaluations secondary to a variety of issues. However, only physicians in whom the referral source reported that the reason for referral was behavior that was inconsistent with behavioral policies and procedures and/or disruptive to the functioning of the system were included in the sample (n = 79). The identified physicians completed a multidisciplinary fitness for duty evaluation that is framed within a biopsychosocial approach and also takes into consideration level of performance within the ABMS six core competency framework. The MicroCog was administered as part of the fitness for duty evaluation to help determine if there were potential health and wellbeing issues that could be contributory. The data used in this study are retrospective. IRB approval for use of the de-identified data comprising the Williams Behavioral Comportment sample was obtained from the Western IRB (Puyallup, WA 98374).

### Instrument

The MicroCog is a computerized neuropsychological screening instrument that assesses neurocognitive functions in adults. It can be administered on almost any laptop computer. The test was released in 1993 and was originally known as the Assessment of Cognitive Skills. The Risk Management Foundation of the Harvard Medical Institutions commissioned the test to screen elderly physicians and other professionals for cognitive impairment. As its original use was to identify impaired physicians, it can detect cognitive deficits in well-educated higher functioning individuals [32]. The MicroCog was selected for use as it has been a widely used clinical tool with outcome measures in a variety of populations, including physicians referred for competency evaluations, National Football League players, and United States Air Force pilots [19, 33, 34].

The test was renamed the MicroCog and made commercially available beginning in 1994. It can be administered in a standard (60 to 90 minutes) or short (30 to 45 minutes) form. There are 18 subtests, which are used to provide summary Indexes at three levels. Level 1 Index scores are “conceptually formed to represent functioning in five respective neurocognitive domains: Attention/Mental Control, Memory, Reasoning/Calculation, Spatial Processing, and Reaction Time.” See Table 1 for a summary of the subtests within each domain. Level 2 Indexes are aggregations of these ability measures to abstract the processing speed and accuracy components of each of the Level 1 Indexes (Information Processing Speed and Information Processing Accuracy). Level 3 Indexes represent global measures of functioning. They differ in the degree of weight given to processing speed: General Cognitive Functioning (weighs speed and accuracy equally), and, General Cognitive Proficiency (preferentially weighs accuracy). Individual subtest scores are computed and are converted to scaled scores (M = 10, SD±3) while Levels 1–3 Index scores are converted to a scale with a mean of 100 and standard deviation of 15 (Standardized scores). The test is nationally normed on a representative sample of 810 adults. The general population panel used for the norm development includes education corrected norms for less than high school, high school, and greater than high school (13–24 years, 15.6 years mean, 16 years median). The sample, is described in detail in the MicroCog manual [30]. The average subtest total score and response time reliability coefficients have a mean of .76 [30, 32]. A comprehensive discussion of the psychometric properties of the instrument can also be found in the manual.

**Table 1:** Table of each subtest in order of presentation, with description and the neurocognitive domain they assess.

Domain	Sub-Test	Test Description
Reaction Time	Timers 1	Reaction time to auditory, visual, and A/V signals
Memory	Address	A name and address are presented
Spatial Processing	Clocks	Seven analog clock faces to be matched with digital time
Memory	Story 1 Immediate Recall	Story is presented; user questioned about details
Reasoning/Calculation	Math	Eight arithmetic problems
Spatial Processing	Tic Tac 1	A 3x3 block pattern presented for immediate reproduction
Reasoning/Calculation	Analogies	Eleven verbal analogy questions
Attention/Mental Control	Numbers Forward	Visual digit span of up to nine digits
Memory	Story 2 Immediate Recall	Similar to Story 1, content changed
Attention/Mental Control	Wordlist 1 Wordlist 2	1: A list of words needing categorization. 2: Discriminating previously presented words from novel words.
Attention/Mental Control	Numbers Reversed	Backward visual digit span
Memory	Address	Multiple choice recognition of prior address
Reasoning/Calculation	Object Match	Multiple choice test for abstraction
Memory	Story 1 Delayed Recall	Delayed recognition of story 1 content
Attention/Mental Control	Alphabet	Random letters to be alphabetized
Spatial Processing	Tic Tac 2	Tic Tac 1 with different content
Memory	Story 2 Delayed Recall	Delayed recognition of story 2 content
Reaction Time	Timers 2	Identical to timers 1

### Comparisons and analysis

Samples employed in this analysis varied as to the number of indexes reported. The MicroCog Norm sample and Powell sample report all 9 Indexes. The Korinek Control and Korinek Medical/Technical samples report 8 Indexes, lacking the General Cognitive Functioning Level 3 index. Both these samples were reported in one study [31]. The Williams Behavioral Comportment sample provides all 9 Indexes. The lack of one Index is not an impediment to the comparison, as the Level 1 Indexes contain the data from all of the assessment subtests, the one Level 3 test not available in one set of data is completely derived from data reported in their entirety in the Level 1 Indexes.

### Statistical software

The analysis was undertaken in a Sample X Index two-way analysis of variance as well as post-hoc paired comparisons. The analysis employed Prism 7 for Mac OS X, version 7.0C, March 1, 2017 (GraphPad Software, Inc., La Jolla, CA 92037 USA).

## Results

Demographics: Demographic characteristics are provided in Table 2. There are no demographic characteristics reported for the Powell sample in the manual.

**Table 2:** Demographic statistics by sample.

	MicroCog Control^1^	Powell Physicians^2^	Korinek Control^3^	Korinek Competency^4^	Williams Comportment
N	810	169	68	267	79
Age (in years)	55.8 (20.9 s.d.)	Not Reported	49.4 (12.5 s.d.)	51.5 (9.1 s.d.)	51.3 (12.1 s.d.)
Sex	405 Female (50.0%)	Not Reported	27 Female (39.7%)	44 Female (16.5%)	21 Female (26.6%)
Race/ Ethnicity	667 Caucasian (82.3%), 87 African American (10.7%), 56 Hispanic (6.9%)	Not Reported	Not Reported	Not Reported	58 Caucasian (67.0%), 18 (19.8%) Not specified
Proceduralist	Not Reported	Not Reported	Not Reported	Not Reported	30%

^1^ Powell, D., Kaplan, E., Whitla, D., Weintraub, S., Catlin, R., & Funkenstein, H. (2004). MicroCog: Assessment of Cognitive Functioning Windows Edition 2004

(MicroCog for Windows): Pearson.

^2^ Powell, D., Kaplan, E., Whitla, D., Weintraub, S., Catlin, R., & Funkenstein, H. (2004). MicroCog: Assessment of Cognitive Functioning Windows Edition 2004

(MicroCog for Windows): Pearson.

^3^ Korinek, L. L. (2005). Neuropsychological differences between physicians referred for competency evaluations and a control group of physicians. Dissertation Abstracts International, 66(5), 2848.

^4^ Korinek, L. L. (2005). Neuropsychological differences between physicians referred for competency evaluations and a control group of physicians. Dissertation Abstracts International, 66(5), 2848.

Physicians in the Behavioral Comportment Sample (n = 79) were referred for issues the referral sources viewed as deviating from policies around professional and workplace behaviors. The sample did not include physicians in whom the primary referral concern was a question about possible issues of impairment related to an alcohol or other substance use disorder or medical condition. Physicians did not self-refer for services, rather were provided feedback that they needed an evaluation. Referral sources included but were not limited to the following: chief of staff, chief medical officer, president of a medical group, vice president of medical affairs, human resource office, medical board and physician health programs. The problematic behaviors included interactions that were described as angry, aggressive, condescending, or non-collegial; lack of timely charting; lack of timeliness to appointments, meetings, and/or surgeries; poor interpersonal boundaries; dual relationships; poor communication with staff, colleagues, trainees and/or patients; and non-compliance with protocols, rules, or monitoring. Compilation of percentages of referrals that fall within these issue categories is misleading as the issues are overlapping, most individuals have several issues cited, the terminology and what behaviors are identified within these categories varies, and even what behavior is identified as most problematic are idiosyncratic to the referral source.

Table 3 provides the reported mean scores and standard deviations from each of the Indexes for each study sample. As noted earlier, the General Cognitive Functioning Index was not reported for the Korinek samples. The calculated means standard deviations are for the Williams Behavioral Comportment sample are reported here as well.

**Table 3:** Means performance, standard deviation, and number of subjects by study and sample.

	MicroCog Norm^1^		Powell Study^2^		Korinek Control^3^		Korinek Medical/ Technical^4^		Williams Behavioral Comportment	
Index	Mean	S.d.	Mean	S.d.	Mean	S.d.	Mean	S.d.	Mean	S.d.
Attention and Mental Control	100	15	105.5	12.2	110.1	9.1	97.82	11.82	105.1	12.4
Reasoning/ calculation	100	15	112.6	12.4	106.9	12.3	99.7	14.17	102.5	14.1
Memory	100	15	110.4	11.1	110.4	10.5	101.43	13.79	108.3	9.7
Spatial processing	100	15	106	9.6	108.8	8.3	99.72	10.87	106.4	10.9
Reaction time	100	15	113.7	11.1	105.9	7.9	104.34	12.21	84.7	12.4
Information Processing Speed	100	15	101.6	13.1	108.9	11	96.55	17.28	103.9	12.2
Information Processing Accuracy	100	15	113.1	10.1	105.9	10.6	98.93	10.99	105.4	12.0
General Cognitive Functioning	100	15	109.3	10.8					105.6	10.9
General Cognitive Proficiency	100	15	108.4	12.7	109.6	9.1	95.83	12.58	105.6	11.9

^1^ Powell, D., Kaplan, E., Whitla, D., Weintraub, S., Catlin, R., & Funkenstein, H. (2004). MicroCog: Assessment of Cognitive Functioning Windows Edition 2004

(MicroCog for Windows): Pearson.

^2^ Powell, D., Kaplan, E., Whitla, D., Weintraub, S., Catlin, R., & Funkenstein, H. (2004). MicroCog: Assessment of Cognitive Functioning Windows Edition 2004

(MicroCog for Windows): Pearson.

^3^ Korinek, L. L. (2005). Neuropsychological differences between physicians referred for competency evaluations and a control group of physicians. Dissertation Abstracts International, 66(5), 2848.

^4^ Korinek, L. L. (2005). Neuropsychological differences between physicians referred for competency evaluations and a control group of physicians. Dissertation Abstracts International, 66(5), 28

**Fig 1:**
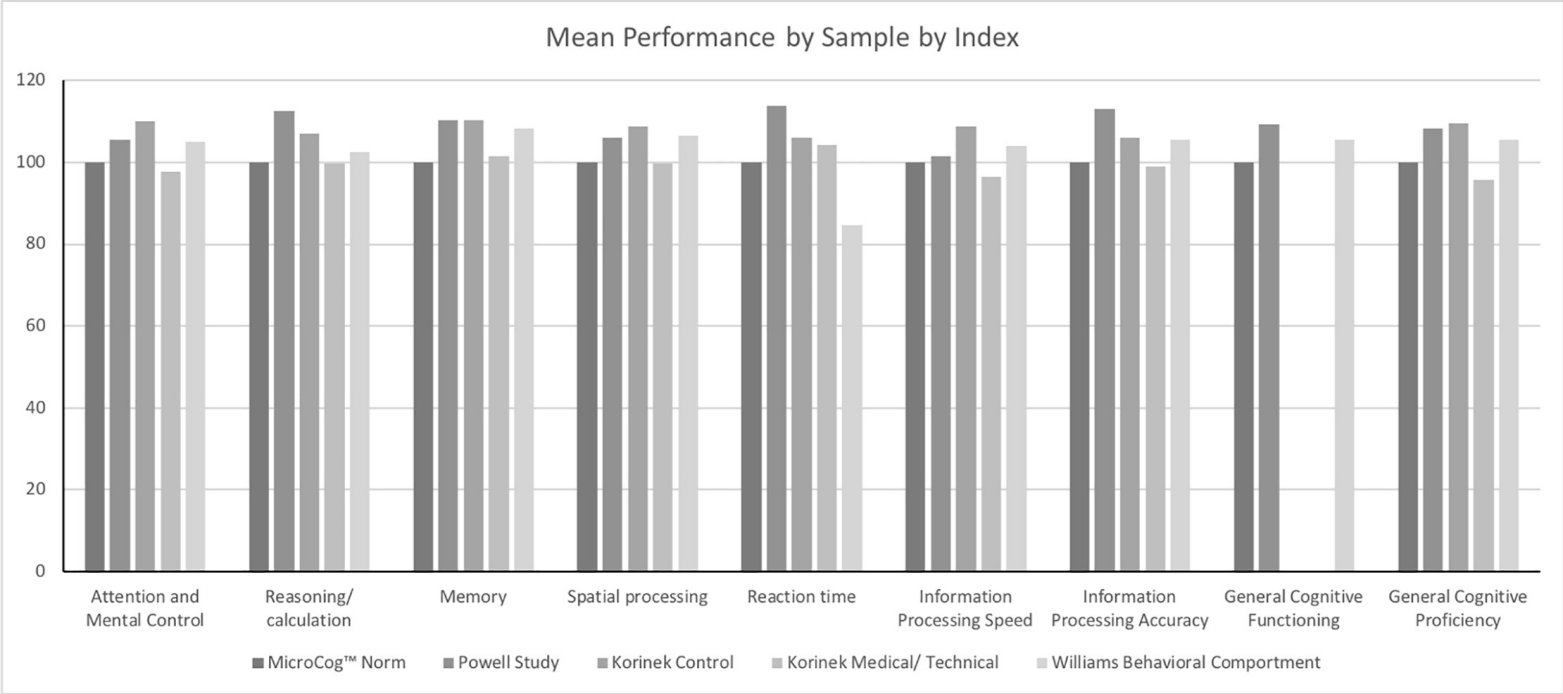
Average performance by data source by index.

Fig 1 provides the Level 1 Indexes in graph form. The data were analyzed across Sample (MicroCog Norm sample, Powell sample, Korinek Control sample, Korinek Medical/Technical sample, and Williams Behavioral Comportment sample). The bars represent mean performance for each Index for each sample. The scores are for each specific MicroCog Index for which all of the samples have the mean and variance available (Attention and Mental Control, Reasoning/Calculation, Memory, Spatial processing, Reaction Time, Information Processing Speed, Information Processing Accuracy, General Cognitive Proficiency). When these data are analyzed using a two-way analysis of variance (Sample x Index), the main effect for both Sample and Index and the interaction are significant (Sample F(4, 11096) = 110.9, p < 0.0001, Index F(7, 11096) = 6.438, p < 0.0001, Interaction F(28, 11096) = 12.78, p < 0.0001.)

**Fig 2:**
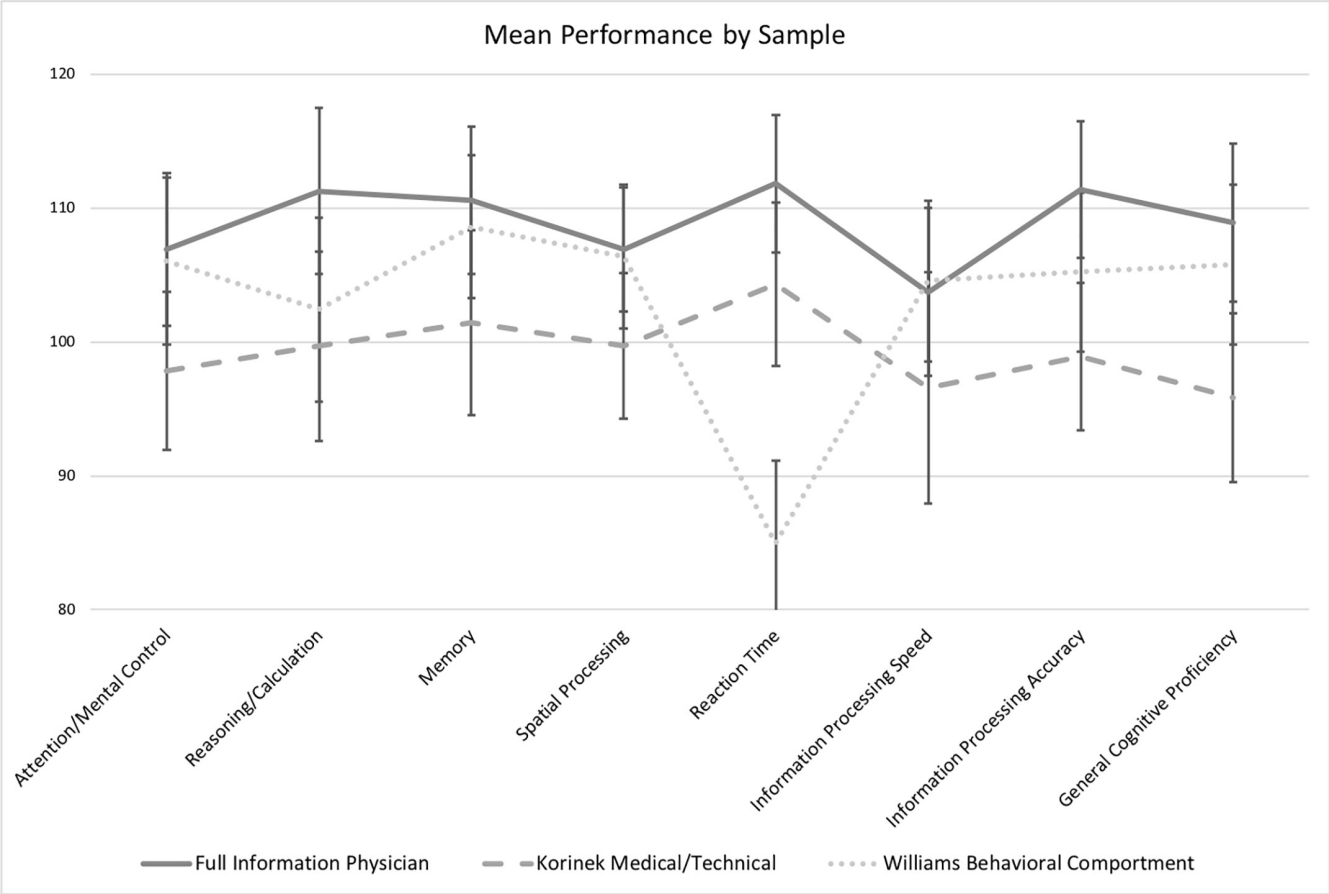
MicroCog normative sample and referred samples performance by index.

To provide a more intuitive test of effect, the two physician normative samples (Powell sample and Korinek Control) were aggregated (Full Information Physician sample). The data were aggregated using a meta-analytic process with unequal variances to generate a full information estimate of the normative performance of the physician population.[26] In the analysis employing the aggregated data a new two-way (Sample X Index) analysis of variance (ANOVA) was significant, (Sample F(2, 4632) = 152.2, p < 0.0001, Index F(7, 4632) = 19.74, p < 0.0001, Interaction F(14, 4632) = 24.27, p < 0.0001.) Fig 2 presents the means of the three samples in graphic form for the Full Information Physician, Korinek Medical/Technical, and Williams Behavioral Comportment samples.

The pattern of differences between the Full Information Physician sample and the two referred physician samples (Korinek Medical/Technical and Williams Behavioral Comportment) was analyzed in a series of pre-planned comparisons to assess differences between groups–the primary focus of interest. First, each referred sample was compared to the Full Information Physician sample employing a Sample X Index 2-way analysis of variance. Each referred sample was statistically different from the Full Information Physician sample. The Korinek Medical/Technical sample was significantly different across Samples (F(1, 4016) = 62.4, p < .0001), across Indexes (F(7, 4016) = 21.45), and the interaction between the two (F(7. 4016) = 4.863. p < .0001). The Williams Behavioral Comportment sample was significantly different across Samples (F(1, 2504) = 102.7, p < .0001), across Indexes (F(7, 2504) = 34.21, p < .0001), and the interaction between the two (F(7, 2504) = 22.01, p < .0001).

The nature of these differences between Indexes across Samples was examined in a series of post-hoc paired comparisons, as no a priori hypotheses were made as to the specific Indexes and differences between samples. In all cases, the post-hoc adjustment was made employing a Bonferroni/Dunn correction. Tables 4 and 5 present comparison data for each Index between the Full Information Physician sample and each of the referred samples, and then between the two referred samples. Comparisons that reach traditional levels of significance after adjustment for post-hoc comparisons are indicated. Fig 2 provides a comparison of sample means across Indexes for these three samples.

**Table 4:** Comparisons of performance by index between samples.

	Full Information Physician (FIP)	Korinek Medical/ Technical (KM/T)—FIP	t ratio	df	Sig.
**Attention/Mental Control**	106.1	-9.093	6.969	4016	**<0.0000001**
**Reasoning/ Calculation**	102.4	-11.57	6.534	4016	**<0.0000001**
**Memory**	108.6	-9.171	8.957	4016	**<0.0000001**
**Spatial Processing**	106.4	-7.217	9.873	4016	**<0.0000001**
**Reaction Time**	84.94	-7.485	2.323	4016	0.1617938
**Information Processing Speed**	104.6	-7.197	6.488	4016	**<0.0000001**
**Information Processing Accuracy**	105.2	-12.46	11.32	4016	**<0.0000001**
**General Cognitive Proficiency**	105.8	-13.09	11.86	4016	**<0.0000001**

**Table 5:** Comparisons of performance by index between samples (continued).

	Williams Behavioral Comportment (WBC)—FIP	t ratio	df	Sig.	KM/T—WBC	t ratio	df	Sig.
**Attention/Mental Control**	-0.8628	0.2502	313	>0.9999999	-8.23	4.732	343	**0.000026**
**Reasoning/ Calculation**	-8.849	2.781	313	**0.0459712**	-2.72	1.537	343	>0.9999999
**Memory**	-1.991	1.949	313	0.4177579	-7.18	4.111	343	**0.0003951**
**Spatial Processing**	-0.5574	2.932	313	**0.0289731**	-6.66	4.772	343	**0.0000217**
**Reaction Time**	-26.89	16.75	313	**<0.0000001**	19.4	12.45	343	**<0.0000001**
**Information Processing Speed**	0.8129	0.1225	313	>0.9999999	-8.01	3.508	343	**0.004091**
**Information Processing Accuracy**	-6.148	4.278	313	**0.0002003**	-6.31	4.478	343	**0.000082**
**General Cognitive Proficiency**	-3.142	2.138	313	0.2663823	-9.95	6.107	343	**<0.0000001**

Each Index comparison between the Korinek Medical/Technical and the Full Information Physician sample was found to be significant with the exception of Reaction Time. The differences in standard scores range from -7.1 for Information Processing Speed to -13.1 for General Cognitive Proficiency. In these cases, the negative prefix indicates lower performance for the Korinek Medical/Technical sample. The range of difference, approximately one-half standard deviation, is the apparent source of the interaction, but all Indexes are depressed in the Korinek Medical/Technical referred sample relative to the Full Information Sample.

By way of contrast, the Williams Behavioral Comportment sample differed significantly from the Full Information Physician sample on only 6 of 8 Indexes reported. The differences range from -6.1 for Information Processing Accuracy, -4.2 for Spatial Processing to -4.4 for Reasoning/Calculation, -3.3 for General Cognitive Proficiency, 3 for Memory, and ending with -22.2 for Reaction Time. The latter, recalling these are standard scores, represents a 2 standard deviation difference. The two other Indexes differ across samples by less than .5 standardized unit (Attention/Mental Control and the Information Processing Speed Index).

When the two referred samples are compared to each other, this pattern becomes even clearer. All save one of the post-hoc comparisons are significant and negative with the exception of Reasoning and Calculation, while the difference on the Reaction Time Index is reversed, indicating that the Korinek Medical/Technical physicians are performing less well, in general, than the Williams Behavioral Comportment physicians. The degree of difference meets typically accepted level of significance even with an adjustment. In the case of Reaction Time, the direction of the difference is reversed with the Williams Behavioral Comportment sample performing significantly more poorly.

We further investigated the Reaction Time Index results. Because the Williams Behavioral Comportment sample is the only sample for which we have detailed, within-subjects data, these results are all developed on data from within that sample. We looked at the subtests Timers 1 –Auditory, Visual, and Cued, and Timers 2 –Auditory, Visual, and Cued. The Timers 1 tests are identical to the Timers 2 tests with the only difference being where in the set of subtests they fall (Timers 2 being administered later in the test battery). The means, presented as MicroCog derived standard scores, are presented in Table 6. The lowest scores are on the Auditory subtests.

**Table 6:** Standardized scores of timers subtests.

	Auditory	Visual	Auditory/Visual
Timers 1	5.31	8.52	11.57
Timers 2	5.36	8.52	11.56

## Discussion

The results of this study support that physicians with workplace performance issues (those with both technical/competency and behavior issues) performed significantly worse on this neuropsychological screening instrument than physicians for whom no such concerns exist. The analysis does not address the cause of this difference. It remains uncertain whether the difference is causal in its relationship to the physicians’ professional dysfunction or merely symptomatic. It is plausible that those in the referred groups have medical, psychiatric, psychological and/or psychosocial factors that contribute both directly to their professional dysfunction as well as their poorer than anticipated neuropsychological performance as such factors have been shown to be associated with possible neuropsychological sequellae.

The more novel findings reported here are that to our knowledge, this is the first study suggesting the possibility of neuropsychological issues in those with behavioral comportment issues, and that those with behavioral comportment issues demonstrate significantly better overall performance than those with medical/technical (clinical competency) issues with the exception of their performance on one subtest. Understanding this difference requires fuller understanding of the causal relationship between neuropsychological function and both medical professionalism as well as medical technical performance.

The performance on the Reaction Time Index for the Behavioral Comportment group was significantly worse relative to all other groups. Inspection of the means indicates that this is primarily due to the auditory component of the test. It is possible that the effect was at least in part a result of age related hearing loss and/or occupationally related hearing loss [35] as approximately 30% of those in the Behavioral Comportment sample were procedural specialists. Another contributory factor could be the degree of ambient noise at the different test sites and/or the volume of the stimuli. Unfortunately, since only mean Index scores were available for each of the other four samples, we were unable to look at patterns of performance on this subtest which would allow for further clarification of this finding. Other measures gathered on physicians in the Behavioral Comportment sample as part of the fitness for duty evaluation indicated that they are highly conscientious. This may have contributed to a slower and more cautious style of responding. This high degree of conscientiousness may also be related to workplace behavioral issues, as individuals with this personality characteristic may want things done a particular way and can have difficulty accommodating changes in routine or approach that may occur within a hospital system particularly when there are members of a multidisciplinary team. Consistent with this explanation, there was a high degree of externalization of blame on the part of referred physicians. Thus, physicians tended to view the problematic behavior in the context of the system’s failure (for example they were provided with poor support staff or the wrong instrument) versus focusing on the problematic nature of their response to potential system issues.

In terms of the broader overall findings of the medical/technical group performing worse than the behavioral comportment group, a number of potential explanations present themselves as worthy of consideration and further investigation. It is possible that as physicians are highly focused on their patients’ medical outcome they compensate through effort for insult to their neuropsychological dysfunction in the domain of medical technical performance more so than they do in other domains of functioning. In this view, by the time medical outcomes deteriorated to the point that physicians were identified as needing remedial intervention, their neuropsychological deficit would be greater than those identified for behavioral issues.

A second, alternative, explanation is that the cognitive burden associated with each of the two functions is different. Behavioral interactions are less scripted, less routinized and less over learned. In this view, the chaotic and unpredictable social environment of interpersonal interactions might cause the physician to underperform with less neuropsychological dysfunction than that required to perturb dysfunction in the more overlearned context of medical technical performance. It is possible, in yet a third view, that these two explanations are both active. In this view, the physician preferentially applies more of his/her resource to the medical technical domain causing dysfunction in the behavioral domain to surface while the neuropsychological dysfunction is still marginal as neurocognitive resources are being diverted to support the medical/technical activities.

It is also possible that the difference relates not to a difference in the sensitivity of the task to neuropsychological dysfunction, but rather to the sensitivity of the observers. In this view the difference would be, in essence, a time of measurement effect. It is possible that given the relative newness, in a social sense, of the Joint Commission’s Sentinel Event 40 [15] that mandated that all Joint Commission approved facilities had policies and procedures in place to address disruptive behavior, and the increased economic power and importance of the allied health professions, interpersonal dysfunction is identified earlier and more aggressively than medical/technical dysfunction. This sensitivity, coupled with the ambiguity of the standard of performance in this domain as compared to medical technical performance, may result in this group of physicians being referred earlier in the process.

Each of these possibilities has significant implications for both physicians’ health and for society. Poor performance on neuropsychological testing can result from medical conditions both reversible and irreversible, psychiatric conditions and/or psychosocial stressors. Clearly, early identification of contributory factors and appropriate treatment is in the physicians’ own best interest as well as society’s best interest to protect patient safety and to protect the resource to society that each physician represents.

For physicians with behavioral comportment issues whose level of performance on the MicroCog approaches the level of the medical/technical group, a prudent least risk approach would be to undertake a more thorough review of available data including any data that addresses possible clinical competency concerns. This might be accomplished through specifically questioning the referral source about indicators of potential clinical competency concerns. If concerns are surfaced, additional evaluation of clinical competency may be warranted. It is also important to recognize that the MicroCog is a screening instrument. Thus, if level of performance on the screening assessment is below expectations relative to available physician norms, consideration of referral for more comprehensive neuropsychological evaluation is warranted.

These results indicate that using typical cutoffs for screening exams, employing the Full Information Physician norm data, yields higher percentages of physicians referred for full neuropsychological testing from the medical/technical group than the behavioral group, see Table 7 (Refer to Williams et al., 2017, [36] for a complete discussion of these norms and recommended cut offs.).

**Table 7:** Percent of failures expected by standard deviations below Williams et Al. generated means.

	Powell Normal	Korinek Normal	Korinek Medical Technical	Williams Comportment
1 Standard Deviation	18.50%	8.30%	53.70%	23.40%
1.5 Standard Deviations	8.50%	2.00%	35.10%	10.90%

Consideration of the potential impact of factors related to health and wellbeing is particularly important as physicians are poor at self-care, issues of mental and physical health are often seen in physicians referred for professionalism issues [37], and physicians have high reported rates of distress, burnout [38] and substance use [39]. Poor neuropsychological performance can be reflective of a number of medical conditions[40], mental health issues such as substance use disorders[40], mood disorders, anxiety disorders, and general distress [10, 41–43]. Thus, the first step in the remediation process for those who have performed below expectations on the screening instrument would be further neuropsychological evaluation to inform next steps. For those whose performance on the screening instrument was at an expected level, comprehensive neuropsychological testing is not indicated; rather a remediation approach guided by the results from other elements of the fitness for duty evaluation would be indicated. This might include coaching, remedial continuing medical education, individual therapy, group therapy or some combination of these resources.

There are a number of limitations in the current study. Demographic data are not available on all of the included samples. Given sample size, we were only able to look meaningfully at main effects and were unable to thoroughly investigate differences across the samples within the indexes. The sample sizes of the groups and the lack of demographic information make it impossible to evaluate for age or specialty-specific differences among the physicians. Of note, however, this analysis utilizes age and education adjusted scores. Age is only a concern if there is an age-by-profession interaction in neuropsychological performance. Additionally, “Age-adjustment of psychological results, even though standard practice, will underestimate the magnitude of any underlying psychological difficulty in absolute terms, and may be less relevant from a quality assurance viewpoint (18).”

Publicly available data from prior published reports were used along with data on physicians referred for behavioral comportment issues. This approach introduces a limitation in that only data available from all of the sources can be used in the analysis, in this case limiting comparisons among groups to means and variances in all preplanned and post hoc comparisons. While data from the physicians in the behavioral comportment sample came from across the United States, the sample is drawn from only one site. This could limit generalizability of the findings. The developers of the MicroCog went to considerable lengths to assure that their normative sample was a reasonable sample of the population as a whole. However, the other samples in this study were samples of convenience. Therefore, we do not know whether the error term is independent and normally distributed across the samples. We also do not know if the performance distribution is, in fact, Gaussian. Thus, it is possible our statistic is biased in some unknown way. The study involves investigating differences between physicians referred to different programs. As the analysis blocks on treatment, the statistic is somewhat less sensitive to sample, but it is possible there is an unknown tertiary causal factor thus there is a possibility that the treatment effect is over or under stated.

## Conclusions

The medical profession continues to be facing ongoing stress due to the expansion of medical knowledge, increasing patient demand, increasing administrative burdens and shortfall of physicians available to meet those needs. The need to maintain currency coupled with increasing burdens suggests that physician performance difficulties will continue to be an issue moving forward. The national rates of burnout[44] and distress, the aging of the medical profession[45, 46], and demand for physician services present challenges to physician health and wellbeing.

The aim of this exploratory study was to examine patterns of performance on a commonly used neuropsychological screening instrument in physicians with identified performance issues, both medical/technical concerns and behavioral comportment concerns who had been referred for either clinical competency evaluations or fitness for duty evaluations. On average, both groups of referred physicians showed significantly poorer performance relative to the physician comparison samples. Those with the poorest neurocognitive performance were physicians referred for deficiencies in medical, cognitive, and procedural skills, while those with behavioral comportment issues performed significantly worse than published norms for comparison physicians but significantly better than the medical/technical group.

While replication of results is warranted, the current results suggest the value of including neurocognitive screening as an element of assessment in physicians referred for any type of performance concern. This includes clinical competency issues and behavioral comportment issues. The MicroCog test may be particularly well-suited to this task as in its initial form it was developed as a screening measure for physicians. There are now preliminary physician norms available[26]. The test also has the advantage that it does not require a skilled psychometrician to administer the test. The current study suggests that further study of the potential role of neurocognitive factors and by extension issues of health and wellbeing in physicians referred for behavioral comportment issues are warranted. This would include exploration of other potential neurocognitive screening instruments, evaluation of the sensitivity and specificity of various neurocognitive screening tests in this population as well as a further evaluation of patterns of neurocognitive performance by reason for referral.

Implications of the current findings at the individual physician level, suggest the benefit of including neuropsychological screening as part of fitness for duty evaluations of physicians referred for any type of performance concern. Further testing following a screening result suggestive of neuropsychological concern would be required to substantiate the findings of the screen and to determine possible patterns and etiologies. This then would inform necessary steps to remediation. This raises the possibility that referring physicians with behavioral comportment issues to some form of remediation such as coaching or remedial CME without some evaluation of neurocognitive screening, may be missing the mark as referral to such services in, absence of other forms of assessment, may be missing potential health and wellbeing issues which could be contributory. From a broader perspective, such an approach would augment traditional medical education frameworks which typically do not focus on identifying the causal factors underlying poor performance[47], particularly in the areas of poor interpersonal and communication skills, lack of professionalism, difficulty accepting feedback or difficulty fitting into the system in which the physician is delivering care.
